# Study on Screening Core Biomarkers of Noise and Drug-Induced Hearing Loss Based on Transcriptomics

**DOI:** 10.1055/s-0043-1777069

**Published:** 2023-12-04

**Authors:** Xin Qiu, Qing-Qing Jiang, Wei-Wei Guo, Ning Yu, Shi-ming Yang

**Affiliations:** 1 Department of Otolaryngology-Head & Neck Surgery, The Sixth Medical Center of PLA General Hospital, National Clinical Research Center for Otolaryngologic Diseases, State Key Lab of Hearing Science, Ministry of Education, Beijing Key Lab of Hearing Impairment Prevention and Treatment Beijing, Beijing, People's Republic of China

**Keywords:** transcriptomics, proteomics, noise, gentamicin, hearing loss, biomarkers

## Abstract

**Background** Noise and drug-induced hearing loss (HL) is becoming more and more serious, but the integration and analysis based on transcriptomics and proteomics are lacking. On the one hand, this study aims to integrate existing public transcriptomic data on noise and gentamicin-induced HL. On the other hand, the study aims to establish the gentamicin and noise-induced HL model of guinea pigs, then to perform the transcriptomic and proteomic analyses. Through comprehensive analysis of the above data, we aim to screen, predict, and preliminarily verify biomarkers closely related to HL.

**Material and Methods** We screened the Gene Expression Omnibus database to obtain transcriptome data expression profiles of HL caused by noise and gentamicin, then constructed the guinea pig HL model and perform the transcriptomic and proteomic analyses. Differential expression and enrichment analysis were performed on public and self-sequenced data, and common differentially expressed genes (DEGs) and signaling pathways were obtained. Finally, we used proteomic data to screen for common differential proteins and validate common differential expression genes for HL.

**Results** By integrating the public data set with self-constructed model data set, we eventually obtained two core biomarkers of HL, which were RSAD2 and matrix metalloproteinase-3 (MMP3). Their main function is to regulate the development of sense organ in the inner ear and they are mainly involved in mitogen-activated protein kinase and phosphoinositol-3 kinase/protein kinase B signaling pathways. Finally, by integrating the proteomic data of the self-constructed model, we also found differential expression of MMP3 protein. This also preliminarily and partially verified the above-mentioned core biomarkers.

**Conclusion and Significance** In this study, public database and transcriptomic data of self-constructed model were integrated, and we screened out two core genes and various signal pathways of HL through differential analysis, enrichment analysis, and other analysis methods. Then, we preliminarily validated the MMP3 by proteomic analysis of self-constructed model. This study pointed out the direction for further laboratory verification of key biomarkers of HL, which is of great significance for revealing the core pathogenic mechanism of HL.

## Introduction

With the industrial and military developments of modern society, the ototoxic substances and noise, which are ubiquitous in the human living environment, have had increasingly adverse effects on human hearing and caused serious hearing loss (HL) problems worldwide. 1 2 3 4 5 6 It is estimated that by 2050, the global incidence of HL will double to more than 900 million people. 7 Although appropriate precautions can minimize relevant exposures, they cannot be completely avoided. Deaf patients are unable to communicate normally with the outside world, and this seriously affects their life quality. Among the many causes of HL, the most common ones are noise and drugs with the latter being mainly caused by aminoglycoside antibiotics, especially gentamicin. 8

Transcriptome sequencing analysis can obtain disease-related biomarkers by analyzing the patient's gene expression profile, thereby providing strong evidence for disease diagnoses and treatments. Currently, there are few reports on integrated transcriptome sequencing analysis for HL. Based on the transcriptomic data of HL caused by noises and gentamicin in the public database, combined with the construction of the guinea pig model of related HL, from which we also obtained transcriptomic and proteomic data, and through bioinformatic methods, we screened, predicted, and initially verified biomarkers closely related to HL, which provides new insights into further mechanism research and clinical treatment of HL.

## Materials and Methods

### Public Database Transcriptome Data Collection and Processing

This study obtained data on gene expression changes in mouse cochlear soft tissue induced by gentamicin and noise from the Gene Expression Omnibus database. The keywords “noise” and “gentamicin” were used to search, respectively. The species included Homo sapiens, guinea pigs, rats, and mice. The data type was high-throughput sequencing or microarray data, with corresponding control groups and experimental groups. The sample size of each group is no less than 3. The final screening selected GSE158431 and GSE8342. The original files of both data sets were downloaded, and the R packages “Affy” and “Simpleaffy” were used to perform background adjustment and normalization processing on the data sets.

### Construction of Animal Model of HL and Collection and Processing of Sequencing Data

#### Construction of Animal Model of HL in Guinea Pigs

The researchers selected 60 healthy guinea pigs of both sexes, with no history of exposure to noise or ototoxic drugs, sensitive acoustic reflexes, and normal hearing in the auditory brainstem response (ABR) audiometry test before the experiment. The 60 guinea pigs were randomly divided into a blank control group, a drug group, and a noise group with 20 animals in each group (40 ears).

The guinea pigs in the drug group were intraperitoneally injected with 50 mg/kg gentamicin every day for 10 consecutive days. The noise group required a binaural pulse noise (50 times, peak sound pressure 163 decibel sound pressure level, pulse width 0.25 ms, frequency 8 k, and interval 6.5 seconds) exposure be performed while the guinea pigs were awake. During the noise exposure period, the temperature and humidity was constant (20–26°C, 45–50%), and the food and water were sufficient. After the noise exposure, the animals were returned to their cages.

After model establishment, audiology (ABR), pathological morphology, and molecular testing and comparative analysis were conducted to confirm that the animal models were established successfully. It was found that compared with the blank group, the remaining groups had varying degrees of functional damage, morphological damage, and molecular changes ( *p* < 0.05).

#### Transcriptomic Sequencing Analysis

##### Material Collection Process

Two cochleae of each guinea pig were taken as one sample. After a guinea pig was decapitated and the cochlea is taken out, the bony volute was quickly peeled off in precooled physiological saline, and all the soft tissues in the cochlea were retained and quickly put into a 1.5-mL Eppendorf (EP) tube, and then stored in a –80°C refrigerator. The whole process was finished within 3 minutes.

##### Sequencing Stage

First, we used a kit to isolate the total ribonucleic acid (RNA) of the sample and detected the integrity and quality of the extracted RNA. Then, we used magnetic beads containing oligo (dT) to enrich the messenger RNA (mRNA) and randomly interrupted the mRNA by adding fragmentation buffer. After that, the first complementary deoxyribonucleic acid (cDNA) strand was synthesized with six-base random hexamers using mRNA as a template. Buffer, deoxynucleotide triphosphates, and DNA polymerase I then were added to synthesize the second cDNA strand. The purified double-stranded cDNA was end-filled, added with A-tail and adapter, and use polymerase chain reaction to amplify the cDNA library. Finally, the Illumina platform was used for on-machine sequencing.

##### Process of Transcriptome Sequencing Off-Machine Data

Fastp software was used to process raw reads in the fastq format, so that the low-quality reads were removed to obtain clean reads for subsequent data analysis. The study used HISAT2 software for reference genome alignment, and obtained the read counts of each gene through HTSeq-count, which ultimately formed the gene expression profile for downstream analysis.

#### Proteomic Sequencing Analysis

##### Material Collection Process

Two cochleae of each guinea pig were taken as one sample. After a guinea pig was decapitated and the cochlea is taken out, the bony volute was quickly peeled off in precooled physiological saline, and all the soft tissues in the cochlea were retained and quickly put into a 1.5-mL EP tube, and then stored in a –80°C refrigerator. The whole process was finished within 3 minutes.

##### Protein Extraction, Concentration Determination, and Sodium Dodecyl-Sulfate Polyacrylamide Gel Electrophoresis Detection

An appropriate amount of frozen sample was taken out and transferred to a 1.5-mL centrifuge tube. The sample was placed in a cold grinding instrument for grinding. The sample lysate and protease inhibitor (phenylmethylsulfonyl fluoride) were added to make it a final concentration of 1 mM. The cold grinding instrument is set at –35°C, 60 Hz, 120 seconds, and repeated once. The solution was centrifuged at 12,000 × *g* at room temperature for 10 minutes, the supernatant taken, and centrifuged again to take the supernatant. The supernatant was the total protein solution of the sample. The protein concentration was determined, aliquoted, and stored at –80°C for later use.

A quantity of 10 μg of protein was used for the separation process using sodium dodecyl-sulfate polyacrylamide gel electrophoresis with a 12% gel. Subsequently, the gel was stained with Kaumas Brilliant Blue using eStain LG (GenScript, Nanjing, China). The analysis of the gel was performed using an automatic digital method.

##### The Sample was Decomposed with Trypsin and the Decomposed Peptide was Desalinated

Based on the recorded protein concentration, extract an equal amount of protein (50μg) from every sample, and then dilute various sets of samples to match in terms of concentration and volume. Mix the protein solution with 25 mM dithiotreitol (DTT), adjusting the volume accordingly, to achieve a final concentration of approximately 5 mM DTT. Then, incubate the mixture at a temperature of 55°C for a duration of 30 minutes. Once cooled to room temperature on ice, proceed by adding the appropriate amount of iodoacetamide to achieve a final concentration of approximately 10 mM. Subsequently, keep the mixture in a dark environment at room temperature for 15 minutes. Next, add six times the amount of prechilled acetone to the aforementioned system to cause protein precipitation, and store it at a temperature of –20°C for a minimum of 4 hours or overnight. Following the occurrence of precipitation, extract the specimen and subject it to centrifugation at a force of 8,000 × *g* for a duration of 10 minutes at a temperature of 4°C. This process aims to gather the sediment, which should then be left undisturbed for a period of 2 to 3 minutes to allow the acetone to evaporate. To dissolve the protein precipitate again, 100 μL of a 50-mM NH4HCO3 solution was added. The corresponding volume of enzymolysis diluent should be added based on the protein content, following a protein-to-enzyme ratio of 50:1 (m/m). For instance, if 100 μg of protein is present, 2 μg of enzyme should be added. Subsequently, the solutions were incubated at 37°C overnight for digestion. The enzymatic reaction was halted by adjusting the pH to 3 using phosphoric acid. Finally, the samples were subjected to desalting using SOLA SPE. Following the vacuum drying process, the samples were reconstituted and supplemented with iRT peptides (1:10).

##### The Samples were Identified by Liquid Chromatography-Tandem Mass Spectrometry

The proteomic data analysis was performed by Shanghai Luming Biological Technology Co., LTD (Shanghai, China). The TimsTOF Pro2 mass spectrometer (Bruker) and nanoElute (Bruker) instruments were employed for conducting shotgun proteomics and data-independent acquisition (DIA) experiments. The samples were loaded onto an EASY-nLCTM 1200 system (Thermo, United States) and separated using a C18 column measuring 25 cm × 75 µm. The flow rate was maintained at 300 nL/min throughout the experiment, while the linear gradient was programmed as follows: from 0 to 45 minutes, a gradient of 2 to 22% B was applied; from 45 to 50 minutes, a gradient of 22 to 37% B was applied; from 50 to 55 minutes, a gradient of 37 to 80% B was applied; and from 55 to 60 minutes, a gradient of 80% B was applied.

In the context of DIA, the collision energy was systematically increased in a linear manner, ion mobility is set from 0.7 to 1.3 Vs/cm ^2^ , with the collision energy varying from 59 to 20 eV, respectively. The tandem mass spectrometry spectra were recorded within the mass-to-charge ratio ( *m* / *z* ) range of 100 to 1,700.

The Spectronaut Pulsar 16.2.221229.55965 (Biognosys, Switzerland) was utilized with its default factory settings for the purpose of conducting a search and generating a library. This process involved the utilization of trypsin/P as the enzyme, allowing for a maximum of two missed cleavages, considering oxidation of Me as a variable modification, carbamidomethyl as a fixed modification, and maintaining a 1% false discovery rate for peptide-spectrum match, peptide, and protein identification. The DIA data was subsequently analyzed using Spectronaut, employing the aforementioned spectral library. Main parameters of the software were set as follows: The unidentified restriction site was observed to occur up to two times. The cysteine residue (C) was stabilized through carbamide methylation modification, while the N-terminal propionylation modification and the oxidation modification of methionine (M) in the protein were subjected to variable modification. To screen the mass spectrometry data, a screening threshold of 0.01 was established for both the mass precursor Q-value and the protein Q-value. The employed data normalization strategy involved local normalization. Our analysis of mass spectrometry data was specifically concentrated on the MS2 level and utilized the uniprot-Cavia porcellus-10141–2023.02.13.fasta database. These experimental parameters and strategies were meticulously selected to align with our study objectives and fulfill our data analysis requirements.

### DEGs Analysis

#### Analysis of DEGs in Public Data Sets

The differentially expressed genes (DEGs) between normal mice and mice treated with noise and gentamicin were screened with the help of the R package-limma. The threshold for DEGs was defined as |LogFC| > 0.5 and *p* -value < 0.05.

#### Analysis of DEGs from Sequencing Data in Self-Constructed HL Model

The study used R language to convert the sequenced data from counts type to tpm type, and then used the limma package to screen the DEGs on the sequenced data of the blank control group, the drug group, and the noise group. The thresholds of DEGs were defined as |LogFC| > 0.5 and *p* -value < 0.05.

### Functional Enrichment Analysis

The online database Metascape was used to conduct functional enrichment analysis of DEGs in public data sets and self-constructed HL model data sets, including Gene Ontology (GO) enrichment, Kyoto Encyclopedia of Genes and Genomes (KEGG), and biomolecular pathway enrichment (Reactome), among which pathways with *p* < 0.05 were considered to be statistically significant.

### Screening of Core Genes and Signaling Pathways for Hearing Damage Caused by Noise and Gentamicin

With the help of the Venn diagram tool to do intersection process of the above data, the common DEGs and signaling pathways of the public data sets and self-constructed HL model data sets for noise and gentamicin-induced HL were obtained.

### Validation of Core Genes for HL Based on Differential Proteomics

Credible proteins and differential proteins can be obtained after DIA quantitative proteomics biological information analysis. Core genes can be obtained through the previous analysis of DEGs. The core genes corresponding to the regulation of differential protein expression can be found in the differential protein data. At the same time, a statistically significant difference was required, which was *p* < 0.05.

### Statistical Method

The statistical analysis involved in this study was completed in R software [V 4.1.3]. The Wilcoxon test was used for comparison between the two sets of data. The standard for statistical significance was a two-sided test, and it was considered statistically significant with *p* < 0.05.

## Results

### Differentially Expressed Gene Analysis

#### Screening of DEGs in Public Data Sets

**Fig. 1:**
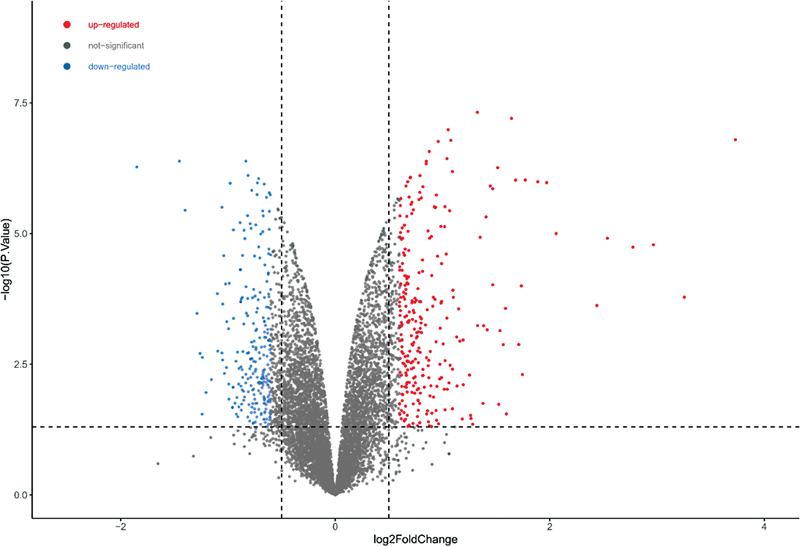
Volcano plot of differentially expressed genes (DEGs) in the public GSE140087 noise data set.

**Fig. 2:**
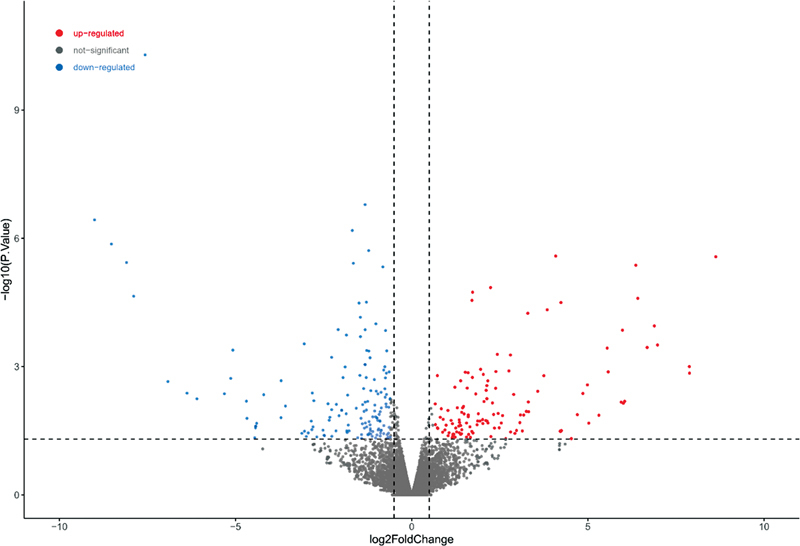
Volcano plot of differentially expressed genes (DEGs) in the public GSE59913 gentamicin data set.

A total of 906 DEGs were screened from the GSE140087 noise data set, including 458 upregulated genes and 448 downregulated genes ( Fig. 1 ). A total of 294 DEGs were screened from the GSE59913 gentamicin data set, including 131 upregulated genes and 163 downregulated genes ( Fig. 2 ).

#### Screening of DEGs from Sequencing Data in Self-Constructed HL Model

**Fig. 3:**
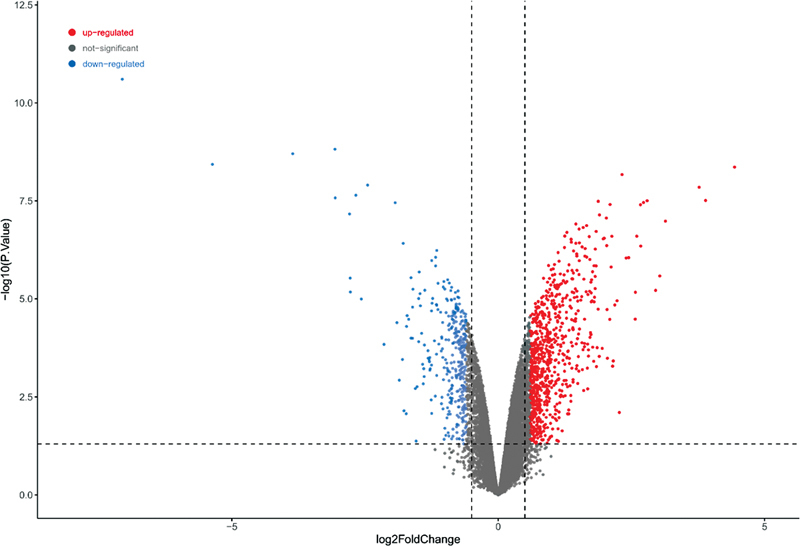
Volcano plot of differentially expressed genes (DEGs) in the noise data set from the self-constructed hearing loss (HL) model.

**Fig. 4:**
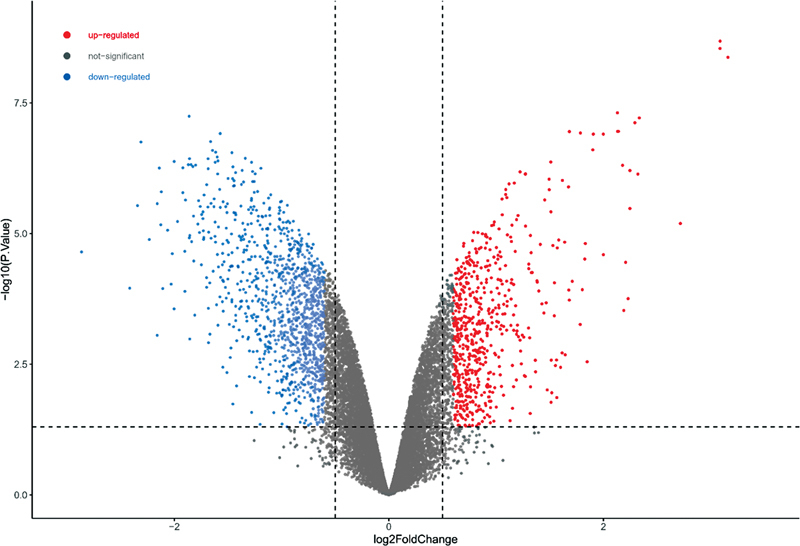
Volcano plot of differentially expressed genes (DEGs) in the gentamicin data set from the self-constructed hearing loss (HL) model

A total of 1,683 DEGs were screened from the noise data set, including 1,159 upregulated genes and 524 downregulated genes ( Fig. 3 ). A total of 2,446 DEGs were screened from the gentamicin data set, including 916 upregulated genes and 1,530 downregulated genes ( Fig. 4 ).

### Functional Enrichment Analysis

#### Functional Enrichment Analysis of DEGs from Public Data Sets

**Fig. 5:**
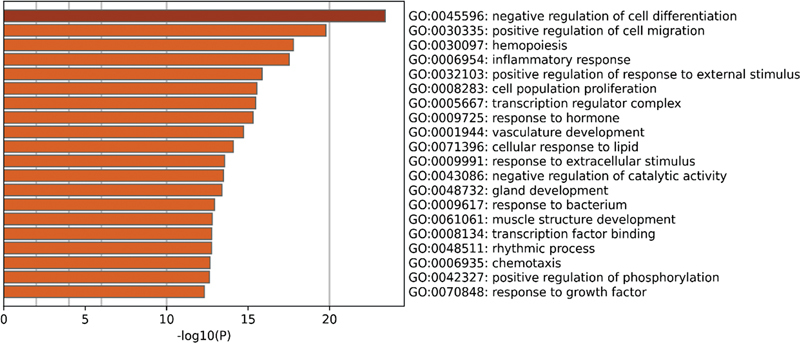
The top 20 pathways enriched by Gene Ontology (GO) of differentially expressed genes (DEGs) in the public GSE140087 noise data set.

**Fig. 6:**
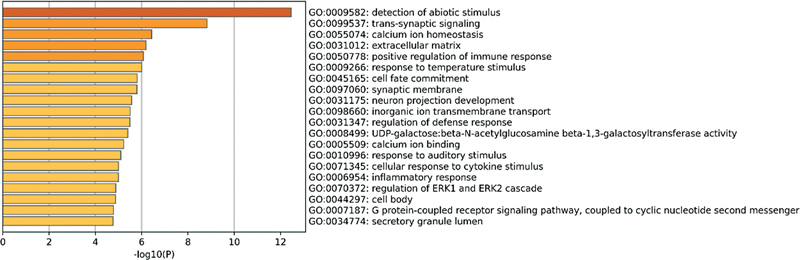
The top 20 pathways enriched by Gene Ontology (GO) of differentially expressed genes (DEGs) in the public GSE59913 gentamicin data set.

In the GSE140087 noise data set, GO enrichment analysis found that these DEGs were mainly enriched in processes such as inflammatory response, cell response to external stimuli, and cell migration ( Fig. 5 ). At the same time, in the GSE59913 gentamicin data set, related DEGs were found to be significantly enriched in aspects such as immune response, inner ear development, and response to external stimuli ( Fig. 6 ).

**Fig. 7:**
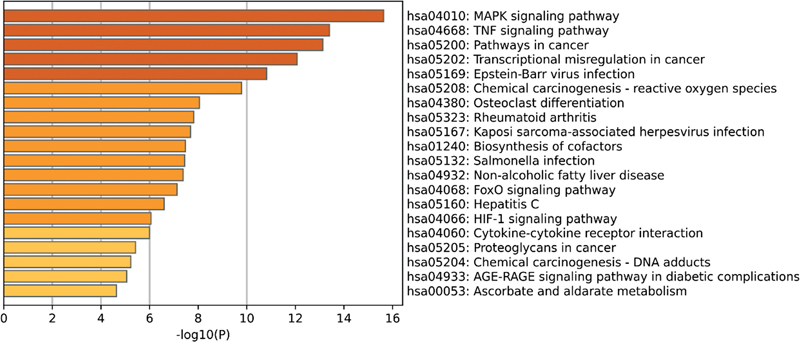
The top 20 pathways enriched by Kyoto Encyclopedia of Genes and Genomes (KEGG) of differentially expressed genes (DEGs) in the public GSE140087 noise data set.

**Fig. 8:**
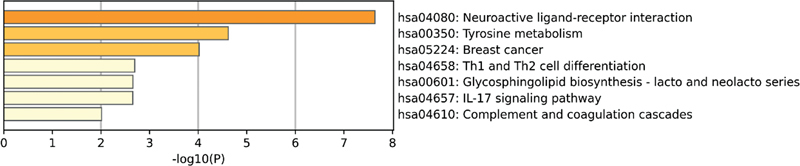
The top 7 pathways enriched by Kyoto Encyclopedia of Genes and Genomes (KEGG) of differentially expressed genes (DEGs) in the public GSE59913 gentamicin data set.

In the GSE140087 noise data set, it was found that the signaling pathways with significant enrichment in KEGG included the mitogen-activated protein kinase (MAPK) signaling pathway, the tumor necrosis factor signaling pathway, and the phosphoinositol-3 kinase/protein kinase B (PI3K-Akt) signaling pathway ( Fig. 7 ). In the GSE59913 gentamicin data set, however, it was mainly enriched in signaling pathways such as neuroactive ligand-receptor interaction and tyrosine metabolism ( Fig. 8 ).

**Fig. 9:**
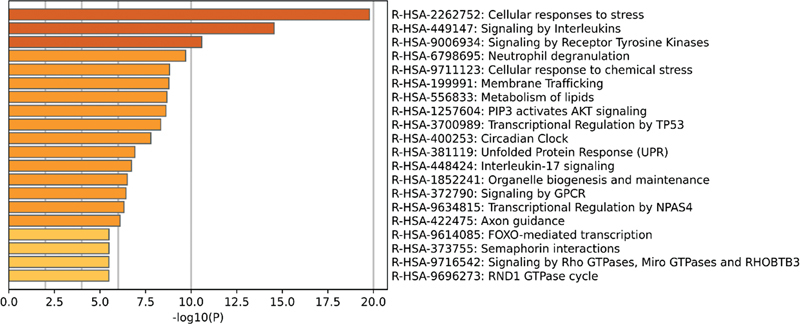
The top 20 pathways enriched in Reactome of differentially expressed genes (DEGs) in the public GSE140087 noise data set.

**Fig. 10:**
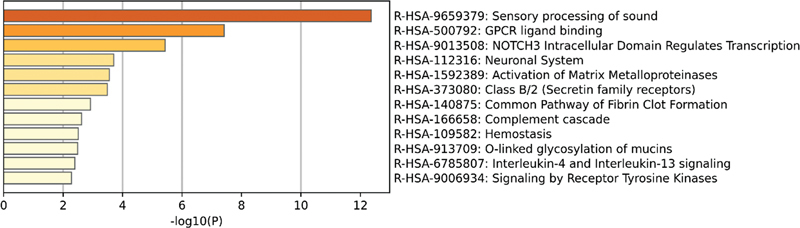
The top 12 pathways enriched in Reactome of differentially expressed genes (DEGs) in the public GSE59913 gentamicin data set.

In addition, a pathway enrichment analysis was also conducted in the Reactome database. The results showed that the DEGs in the noise injury data set were mainly enriched in signaling pathways related to metabolism and Toll-like receptors ( Fig. 9 ), while gentamicin-induced hearing impairment DEGs were significantly enriched in sensory perception, sensory processing of sound, and G protein-coupled receptor (GPCR) signaling ( Fig. 10 ).

#### Functional Enrichment Analysis of DEGs from Sequencing Data in Self-Constructed HL Model

**Fig. 11:**
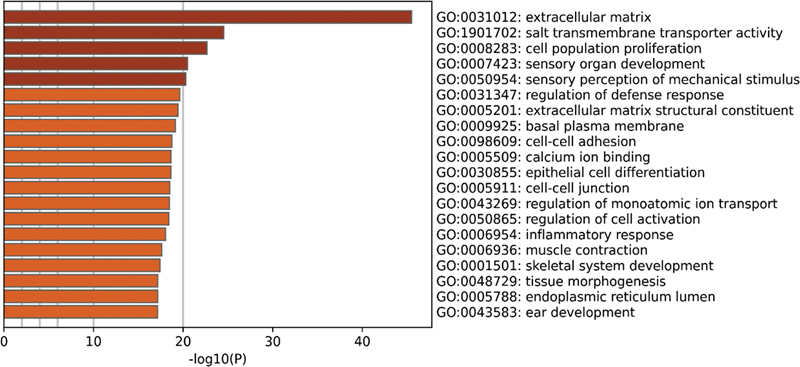
The top 20 pathways enriched by Gene Ontology (GO) of differentially expressed genes (DEGs) in the noise data set from the self-constructed hearing loss (HL) model.

**Fig. 12:**
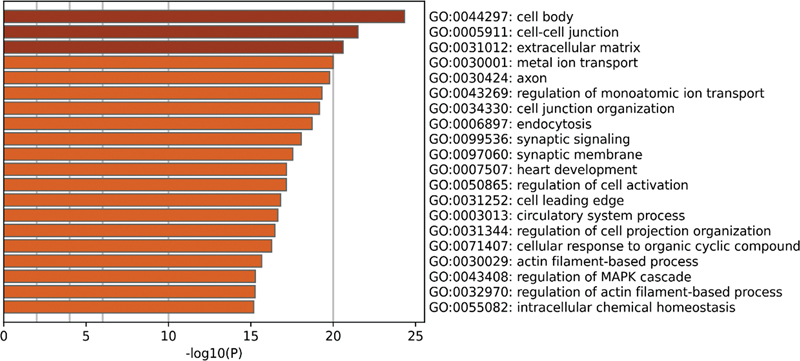
The top 20 pathways enriched by Gene Ontology (GO) of differentially expressed genes (DEGs) in the gentamicin data set from the self-constructed hearing loss (HL) model.

In the noise data set from the self-constructed HL model, GO enrichment analysis results showed that these DEGs were mainly significantly enriched in the extracellular matrix, sensory perception of mechanical stimulation, sensory organ development, inflammatory response, and sensory perception of sound ( Fig. 11 ), while the DEGs in the gentamicin data set from the self-constructed HL model were mainly enriched in GO functions, such as extracellular matrix, regulation of MAPK cascade, sensory organ development, and cellular molecular transport ( Fig. 12 ).

**Fig. 13:**
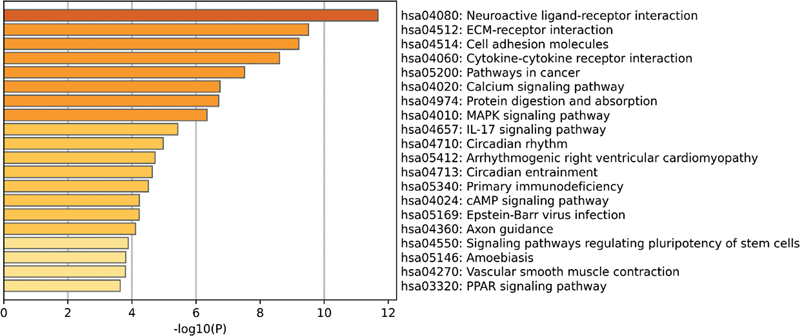
The top 20 pathways enriched by Kyoto Encyclopedia of Genes and Genomes (KEGG) of differentially expressed genes (DEGs) in the noise data set from the self-constructed hearing loss (HL) model.

**Fig. 14:**
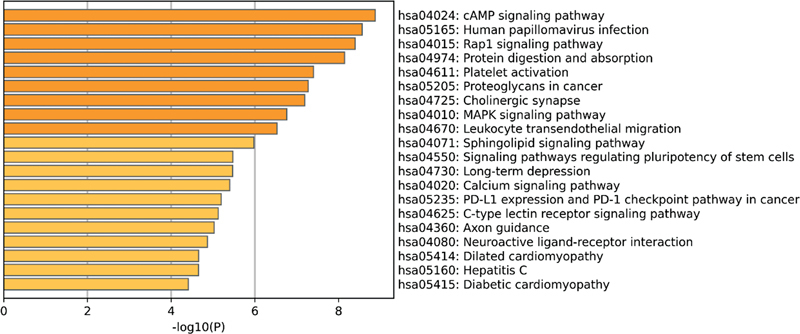
The top 20 pathways enriched by Kyoto Encyclopedia of Genes and Genomes (KEGG) of differentially expressed genes (DEGs) in the gentamicin data set from the self-constructed hearing loss (HL) model.

The enrichment analysis of KEGG signaling pathway showed that DEGs in the noise data set from the self-constructed HL model were significantly enriched in neuroactive ligand-receptor interactions, PI3K-Akt, cytokine-cytokine receptor interactions, MAPK, and peroxisome proliferator-activated receptor ( Fig. 13 ). The DEGs in the gentamicin data set from the self-constructed HL model were mainly enriched in Rap1, MAPK, PI3K-Akt, and cGMP-PKG ( Fig. 14 ).

**Fig. 15:**
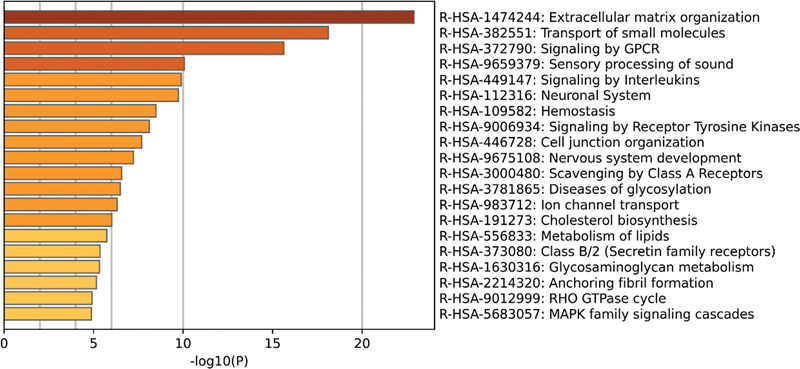
The top 20 pathways enriched in Reactome of differentially expressed genes (DEGs) in the noise data set from the self-constructed hearing loss (HL) model.

**Fig. 16:**
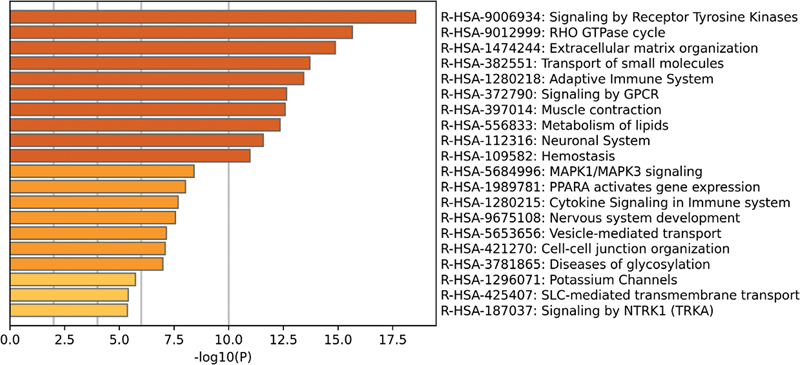
The top 20 pathways enriched in Reactome of differentially expressed genes (DEGs) in the gentamicin data set from the self-constructed hearing loss (HL) model.

In addition, the signal pathway enrichment analysis in the Reactome database showed that the DEGs in the noise data set from the self-constructed HL model was mainly concentrated in GPCR signaling, sensory processing of sound, interleukin signaling, sensory processing of sound by cochlea, and MAPK family signal cascade ( Fig. 15 ). The DEGs in the gentamicin data set from the self-constructed HL model were mainly enriched in receptor tyrosine kinase signaling, GPCR signaling, MAPK family signaling cascade, and vascular endothelial growth factor signaling ( Fig. 16 ).

### Screening of Core Genes and Signaling Pathways for Single-Factor HL in Guinea Pigs

**Fig. 17:**
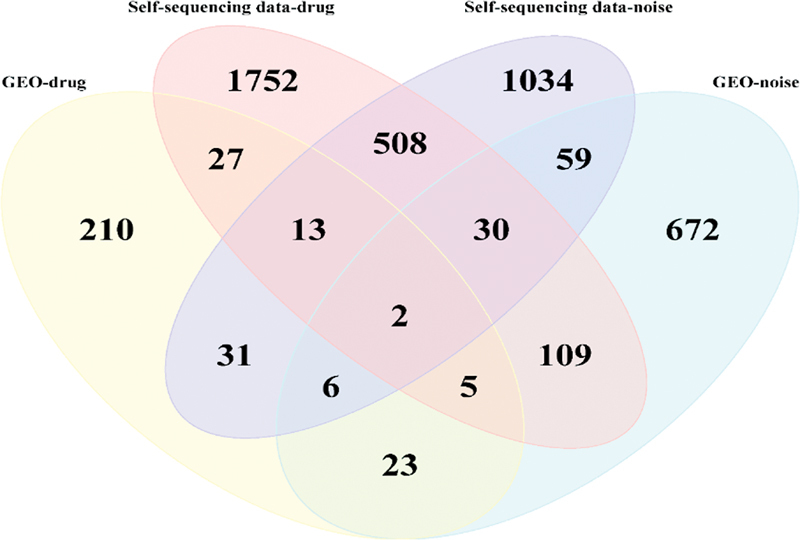
Venn diagram of differentially expressed genes (DEGs) between the public data sets and data sets from self-constructed hearing loss (HL) model.

**Fig. 18:**
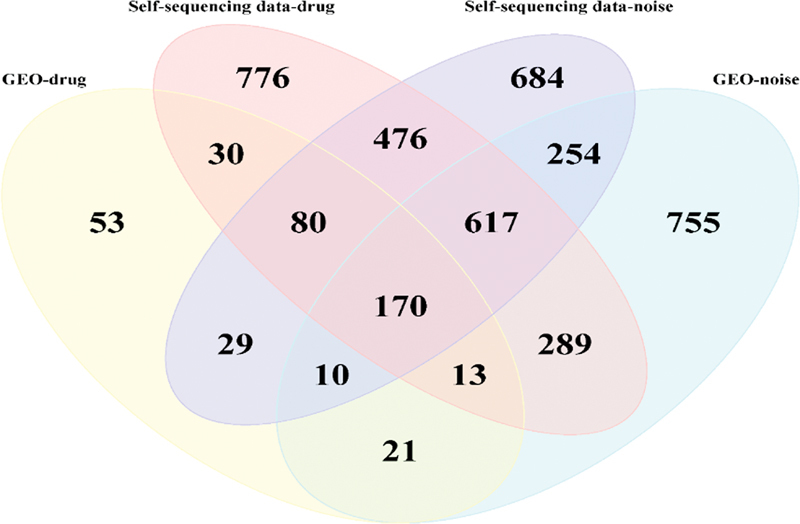
Venn diagram of differentially expressed genes (DEGs) related to Gene Ontology (GO) enrichment pathways between the public data sets and data sets from self-constructed hearing loss (HL) model.

**Fig. 19:**
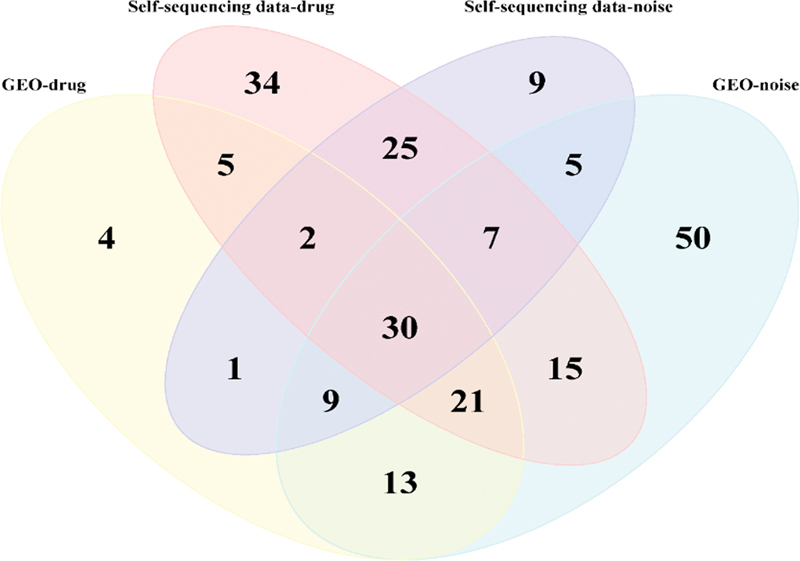
Venn diagram of differentially expressed genes (DEGs) related to Kyoto Encyclopedia of Genes and Genomes (KEGG) signaling pathways between the public data sets and data sets from self-constructed hearing loss (HL) model.

**Fig. 20:**
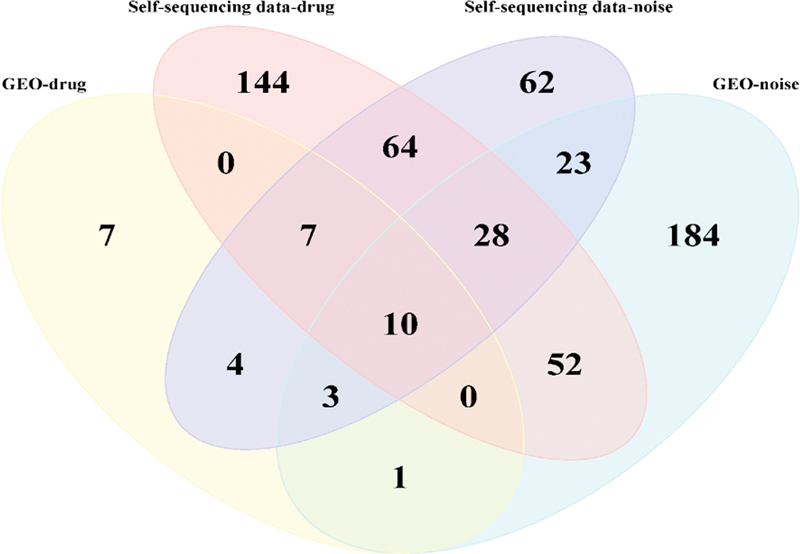
Venn diagram of differentially expressed genes (DEGs) related to Reactome-enriched signaling pathways between the public data sets and data sets from self-constructed hearing loss (HL) model.

The DEGs and enrichment results in the public data set and self-constructed HL model data set were integrated and intersected. What was obtained included two core biomarkers (RSAD2 and matrix metalloproteinase-3 [MMP3]) ( Fig. 17 ), 170 GO-related enrichment pathways ( Fig. 18 ), 37 KEGG-related signaling pathways ( Fig. 19 ), and 10 Reactome-enriched signaling pathways ( Fig. 20 ). The core GO enrichment functions of DEGs included inner ear development, sensory organ development and cartilage development, and the core regulatory signaling pathways of DEGs included MAPK, PI3K-Akt, receptor tyrosine kinase signaling, GPCR signaling, and Toll-like receptors.

### Validation of Core Genes Based on Proteomics of Self-Constructed HL Model

**Fig. 21:**
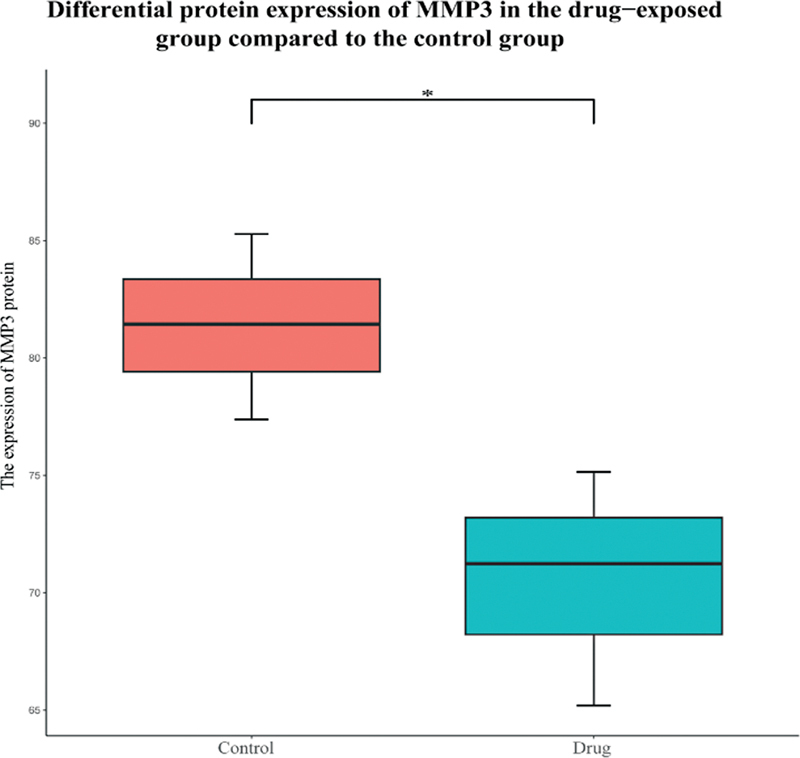
Differential expression of matrix metalloproteinase-3 (MMP3) protein between drug and control group in the self-constructed model. * indicates *p* < 0.05; ** indicates *p* < 0.01.

**Fig. 22:**
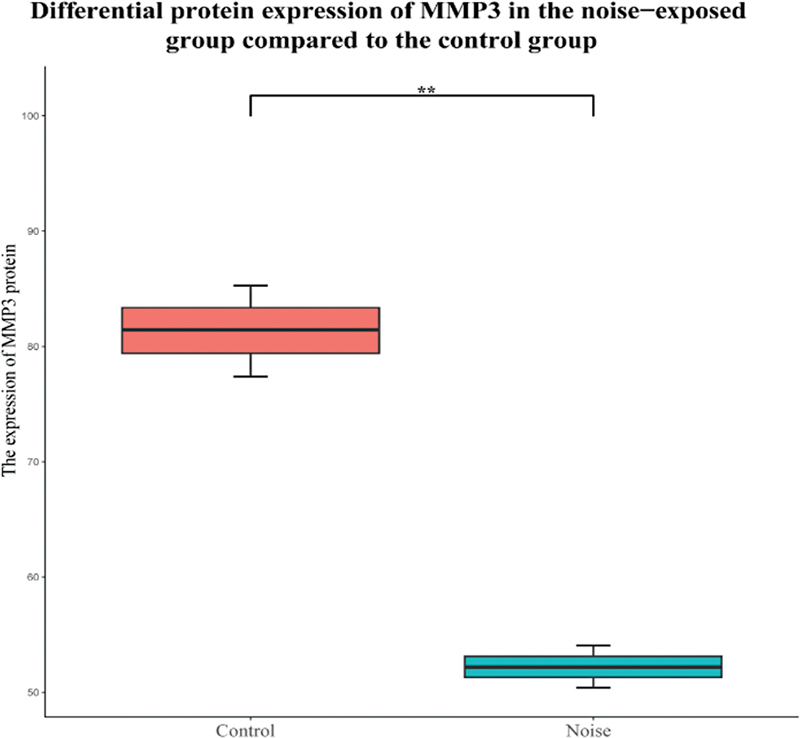
Differential expression of matrix metalloproteinase-3 (MMP3) protein between noise and control group in the self-constructed model. * indicates *p* < 0.05; ** indicates *p* < 0.01.

This study combined the proteomic data set for preliminary verification. The result showed that the expression level of MMP3 and its corresponding protein were significantly different between animals with HL and healthy individuals ( *p* < 0.05) ( Figs. 21 and 22 ).

## Discussion

With the continuous development of modern society, ototoxic substances or noise are widely present in our daily life, resulting in increasingly serious HL problems. In the United States, acquired HL has become the third most common chronic physical disease after high blood pressure and arthritis. It is estimated that more than 900 million people worldwide will suffer from HL by 2050, yet no effective prevention or treatments have been identified.

Transcriptome sequencing analysis technology, which is relatively mature today, can obtain relevant biomarkers by analyzing patients' gene expression profiles, thereby providing new strategies for disease diagnosis and treatments. This study integrates the public database data and transcriptomics and proteomics data of the self-built guinea pig HL model. We jointly analyzed the data obtained in two different ways to obtain biomarkers related to HL, so as to provide new ideas for further laboratory verification of the mechanism of HL.

By integrating the public database and the transcriptome sequencing data of self-constructed HL model, two core genes for HL and multiple signaling pathways were obtained through screening.

The RSAD2 gene, also known as viperin, plays a vital role in the human immune system. It has been shown as a gene associated with HL, 9 which can be used to diagnose and treat patients with HL. This was also proved by the result of our study. MMP3, called stromalin-1, is encoded by the MMP3 gene and is involved in the breakdown of extracellular matrix proteins. Previous research has shown that HL in patients with rheumatoid arthritis is linked to damage to inner ear hair cells by MMP3 through oxidation. 10 At the same time, Zhang et al 11 found that the expression level of MMP3 in Alzheimer's patients with HL was significantly elevated by exploring the clinical features and potential mechanisms related to pathological neuromarkers and blood-brain barrier in Alzheimer's disease and HL, which also confirmed that the expression of MMP3 gene is closely related to HL. In addition, evidence also proved that treatment with MMP3 inhibitors protects hair cells and reduces permanent threshold shifts, suggesting that excessive expression of the MMP3 gene may induce cochlear damage. 12

The MAPK signaling pathway regulates cell growth, differentiation, proliferation, apoptosis, and migration by transmitting extracellular stimulus signals into cells and their nuclei. Zhang et al 13 found that, by injecting superoxide dismutase 2 (SOD2) and gene therapy in mice, when SOD2 is overexpressed, it can counteract the effects of noise and ototoxic drugs and improve the symptoms of HL, and PI3K and MAPK signaling pathways are also affected by SOD2, indicating that modulating PI3K and MAPK signaling pathways may have some therapeutic effects on HL. In addition, Alagramam et al 14 through sequencing and bioinformatics analysis of three groups of mice exposed to different noises, found that except for the control group, the MPAK signaling pathway of mice exposed to different noise decibels in both groups was activated to varying degrees, and finally the analysis pointed out that MAPK signaling is the main pathway for various types of HL, and future treatments and drugs based on the MAPK pathway may play an important role in protecting HL caused by ototoxic drugs. 15 There is substantial evidence that a reduction in PI3K-Akt signaling pathway after various injuries and stimuli is associated with HL and hair cell death. 16 Zhang et al 17 investigated the effects of epigallocatechin-3-gallate (EGCG) on the proliferation and neurosphere formation of neural stem cells (NSCs) in isolated mouse cochlea and found that EGCG promotes cell growth and neuronal differentiation in cochlear NSCs through the PI3K-Akt signaling pathway, which may be used in the treatment of HL. Interestingly, we also found the calcium signaling pathway in the enrichment of signaling pathways in numerous data sets. Calcium is one of the important cofactors involved in apoptosis-degrading enzymes and is essential for cell survival, and numerous studies have shown that calcium channel blockers (CCBs) can effectively prevent damage to cochlear cells, and a recent study has demonstrated that systemic and intratympanic (direct) application of CCBs can prevent HL in cisplatin and noise-induced ototoxicity models. 18 Lai et al 19 found that the calcium protease inhibitor MDL-28170 not only prevented noise-induced synapse loss in hair cells, but also reduced noise-induced damage to the cochlea's sensory hair cells, it was also able to prevent noise-induced hair cell death and HL by upregulating PI3K-Akt cell survival signaling pathways.

Core genes and key signaling pathways are the key areas of diagnosis and treatment of HL patients, which is also a new direction that needs to be explored in the future to prevent or treat HL.

## Conclusion

In this study, two core biomarkers of HL and multiple key signaling pathways were screened and obtained through the analysis of the public database and self-constructed model database. RASD2 and MMP3, as the core biomarkers of HL, can alleviate the symptoms of HL patients by promoting or inhibiting their expression. The regulation of MAPK signaling pathway, PI3K-Akt signaling pathway, and calcium signaling transduction plays an important role in the survival of auditory hair cells. This can provide new ideas for further exploration of the mechanism of HL and further elucidation of the occurrence and development mechanism of HL. It would also provide new ideas and directions for clinical treatment of HL. However, this study still has certain limitations, and the core biomarkers and key signaling pathways of HL obtained in this study still need to be further verified in the laboratory, and our team would continue to carry out experiments on core biomarkers and key signaling pathways.

## References

[1] Kil J Harruff E E Longenecker R J Development of ebselen for the treatment of sensorineural hearing loss and tinnitus Hear Res 2022 413 202 108209 33678494 10.1016/j.heares.2021.108209

[2] Carlson K Schacht J Neitzel R L Assessing ototoxicity due to chronic lead and cadmium intake with and without noise exposure in the mature mouse J Toxicol Environ Health A 2018 81 20 1041 1057 30239325 10.1080/15287394.2018.1521320 PMC6349363

[3] Fetoni A R Rolesi R Paciello F Styrene enhances the noise induced oxidative stress in the cochlea and affects differently mechanosensory and supporting cells Free Radic Biol Med 2016 101 211 225 27769922 10.1016/j.freeradbiomed.2016.10.014

[4] Chen G D Chi L H Kostyniak P J Henderson D Styrene induced alterations in biomarkers of exposure and effects in the cochlea: mechanisms of hearing loss Toxicol Sci 2007 98 01 167 177 17420221 10.1093/toxsci/kfm078

[5] Chen G D Henderson D Cochlear injuries induced by the combined exposure to noise and styrene Hear Res 2009 254(1-2):25 33 19371775 10.1016/j.heares.2009.04.005

[6] Li H Steyger P S Synergistic ototoxicity due to noise exposure and aminoglycoside antibiotics Noise Health 2009 11 42 26 32 19265251 10.4103/1463-1741.45310 PMC2713742

[7] Rosati R Jamesdaniel S Environmental exposures and hearing loss Int J Environ Res Public Health 2020 17 13 4879 32645823 10.3390/ijerph17134879 PMC7370016

[8] Zhao Y Jiang J Chen X M The kinds of ototoxic drugs and the mechanism of damage to inner ear and its prevention Med J Nat Defending Force Southwest China 2019 40 11 718 723

[9] Lei M Zhang D Sun Y Web-based transcriptome analysis determines a sixteen-gene signature and associated drugs on hearing loss patients: a bioinformatics approach J Clin Lab Anal 2021 35 12 e24065 34758154 10.1002/jcla.24065 PMC8649328

[10] Nasution M ES Haryuna T SH Elevated matrix metalloproteinase-3 level may affect hearing function in patients with rheumatoid arthritis J Chin Med Assoc 2019 82 04 272 276 30893257 10.1097/JCMA.0000000000000036

[11] Zhang W J Li D N Lian T H Clinical features and potential mechanisms relating neuropathological biomarkers and blood-brain barrier in patients with Alzheimer's disease and hearing loss Front Aging Neurosci 2022 14 911028 35783139 10.3389/fnagi.2022.911028 PMC9245454

[12] Park J S Kang S J Seo M K Jou I Woo H G Park S M Role of cysteinyl leukotriene signaling in a mouse model of noise-induced cochlear injury Proc Natl Acad Sci U S A 2014 111 27 9911 9916 24958862 10.1073/pnas.1402261111 PMC4103370

[13] Zhang Y Huang S Dai X SOD2 alleviates hearing loss induced by noise and kanamycin in mitochondrial DNA4834-deficient rats by regulating PI3K/MAPK signaling Curr Med Sci 2021 41 03 587 596 34169429 10.1007/s11596-021-2376-4

[14] Alagramam K N Stepanyan R Jamesdaniel S Chen D H Davis R R Noise exposure immediately activates cochlear mitogen-activated protein kinase signaling[J]Noise Health 2014 16 73 400 409 25387536 10.4103/1463-1741.144418 PMC4591925

[15] Liu Y Wei M Mao X Chen T Lin P Wang W Key signaling pathways regulate the development and survival of auditory hair cells Neural Plast 2021 2021 5.522717E6 10.1155/2021/5522717 PMC8214483 34194486

[16] Chen J Yuan H Talaska A E Hill K Sha S H Increased sensitivity to noise-induced hearing loss by blockade of endogenous PI3K/Akt signaling J Assoc Res Otolaryngol 2015 16 03 347 356 25790950 10.1007/s10162-015-0508-x PMC4417093

[17] Zhang Y He Q Dong J Jia Z Hao F Shan C Effects of epigallocatechin-3-gallate on proliferation and differentiation of mouse cochlear neural stem cells: Involvement of PI3K/Akt signaling pathway Eur J Pharm Sci 2016 88 267 273 27012759 10.1016/j.ejps.2016.03.017

[18] Naples J G Calcium-channel blockers as therapeutic agents for acquired sensorineural hearing loss Med Hypotheses 2017 104 121 125 28673569 10.1016/j.mehy.2017.05.036

[19] Lai R Fang Q Wu F Pan S Haque K Sha S H Prevention of noise-induced hearing loss by calpain inhibitor MDL-28170 is associated with upregulation of PI3K/Akt survival signaling pathway Front Cell Neurosci 2023 17 1.199656E6 10.3389/fncel.2023.1199656 PMC10359991 37484825

